# A Two-Stage Multistep-Ahead Electricity Load Forecasting Scheme Based on LightGBM and Attention-BiLSTM

**DOI:** 10.3390/s21227697

**Published:** 2021-11-19

**Authors:** Jinwoong Park, Eenjun Hwang

**Affiliations:** School of Electrical Engineering, Korea University, 145 Anam-ro, Seongbuk-gu, Seoul 02841, Korea; timeless@korea.ac.kr

**Keywords:** smart grid, electricity load forecasting, multistep-ahead forecasting, light gradient boosting machine, attention mechanism

## Abstract

An efficient energy operation strategy for the smart grid requires accurate day-ahead electricity load forecasts with high time resolutions, such as 15 or 30 min. Most high-time resolution electricity load prediction techniques deal with a single output prediction, so their ability to cope with sudden load changes is limited. Multistep-ahead forecasting addresses this problem, but conventional multistep-ahead prediction models suffer from deterioration in prediction performance as the prediction range is expanded. In this paper, we propose a novel two-stage multistep-ahead forecasting model that combines a single-output forecasting model and a multistep-ahead forecasting model to solve the aforementioned problem. In the first stage, we perform a single-output prediction based on recent electricity load data using a light gradient boosting machine with time-series cross-validation, and feed it to the second stage. In the second stage, we construct a multistep-ahead forecasting model that applies an attention mechanism to sequence-to-sequence bidirectional long short-term memory (S2S ATT-BiLSTM). Compared to the single S2S ATT-BiLSTM model, our proposed model achieved improvements of 3.23% and 4.92% in mean absolute percentage error and normalized root mean square error, respectively.

## 1. Introduction

A smart grid is an efficient and intelligent energy operation solution that integrates traditional power systems and information and communications technology to enable two-way communication between consumers and suppliers [1]. Typical smart grids utilize renewable energy resources such as solar photovoltaic and wind power, and operate an energy management system to establish efficient energy operation plans through short-term energy load and supply forecasting. In particular, to establish elaborate energy operation strategies, a high time resolution, such as 15 or 30 min, is essential in short-term load forecasting (STLF) [2]. Furthermore, STLF can be used to establish peak load response or energy storage system operation strategies to reduce energy costs. STLF is becoming increasingly important as it contributes to the implementation of stable power systems through the proper balance between supply and demand [3,4].

So far, many STLF models have been proposed based on various methods [5]. For instance, statistical models for STLF include autoregressive [6], regression analysis [7], and holt winters [8]. These models perform well when the input and output are linear, but degrade when the input and output are nonlinear [9,10]. To address this concern, artificial intelligence (AI)-based models such as support vector regression (SVR) [11], neural networks (NN) [12], and extreme learning machines (ELMs) [13] have been proposed for single-point prediction. Even though such single-output forecasting models perform well even for nonlinear data, they show limited ability to cope with sudden electricity load changes [14]. To compensate for this, various multistep-ahead (MSA) forecasting models have been proposed using recurrent neural networks (RNNs), such as long short-term memory (LSTM) [15] and gated recurrent unit (GRU) [16], that reflect time series characteristics satisfactorily. The performance was further improved by using a sequence-to-sequence (S2S) architecture combining an encoder RNN and a decoder RNN [17]. However, information loss problems arose in S2S architecture RNN models because the encoder compresses all necessary information into a fixed-length vector. To tackle this problem, attempts have been made to apply an attention mechanism to forecasting models so that the decoder can look back on the most relevant information from the encoder output [18,19]. However, this approach showed performance deterioration as the forecasting horizon was expanded [20].

In this paper, we propose a two-stage MSA electricity load forecasting model to deal with the aforementioned issue. The forecasting horizon is one day from the current time at 15 min intervals, which gives 96 time points. In the first stage, a single-output forecasting model performs day-ahead prediction. The forecasting model is constructed using light gradient boosting machine (LightGBM) and time-series cross-validation (TSCV). In the second stage, an MSA forecasting model based on an S2S bidirectional LSTM with attention mechanism (ATT-BiLSTM) performs predictions for 96 time points using the output of the first-stage forecasting model and other external data. The contributions of this paper are as follows:

- We present a forecasting model that combines an ensemble learning method and an RNN for accurate MSA forecasting;
- We show that the performance of an MSA forecasting model can be further improved by considering the prediction result of a single-output forecasting model;
- The proposed model shows very stable forecasting accuracy over the entire forecasting horizon of 96 time points at 15 min intervals.

This paper is organized as follows. Section 2 introduces some related works. Section 3 describes data collection and preprocessing. Section 4 presents the overall structure of the proposed two-stage MSA forecasting model. Section 5 illustrates the experiments and their results. Finally, Section 6 concludes the paper.

## 2. Related Works

Recently, forecasting models using AI methods such as artificial neural networks (ANNs) [21,22] and SVR [23] have been proposed to overcome nonlinearities and complex relationships of time series. For instance, Grolinger et al. [24] constructed a forecasting model based on ANN and SVR to accurately forecast the electricity load of an entertainment building. Jurado et al. [25] predicted the hourly electrical loads of three teaching buildings using various machine learning (ML) methods such as random forest (RF), ANN, fuzzy inductive reasoning (FIR), and autoregressive integrated moving average (ARIMA). Zhang et al. [26] proposed an SVR-based electricity load forecasting model and optimized the hyperparameters using the Cuckoo search algorithm.

On the other hand, Zheng et al. [27] proposed an LSTM-based short-term electricity load forecasting model and showed that an LSTM-based forecasting model can predict complex univariate electricity loads with non-stationarity and non-seasonality. Marino et al. [28] also constructed two electricity load forecasting models based on the standard LSTM and the S2S architecture LSTM, and compared their performance. Both models were trained and tested for one-hour and one-minute time resolution data. They showed that the standard LSTM was unable to accurately forecast loads for one-minute resolution, but the S2S architecture LSTM performed well for one-hour and one-minute time resolution datasets. 

Even though these single-output forecasting models have demonstrated good prediction accuracy, they are limited in handling uncertainties, such as sudden load changes. To address this issue, various MSA models have been proposed by simultaneously predicting multiple time points. For instance, Kim et al. [29] proposed a recurrent inception convolution neural network (RICNN) for MSA electricity load forecasting (48 time points at 30 min intervals). They combined the RNN and the 1-D convolution inception to calibrate the forecasting time and hidden state vector values calculated from the nearby forecasting time points. Jung et al. [30] proposed an MSA load forecasting model (24 time points at 1 h intervals) using a GRU network with attention mechanism. They demonstrated that an attention mechanism can improve the forecasting performance of RNNs by 10% or more. Kuo et al. [31] proposed a one-dimensional CNN-based power load prediction model with three convolution layers. Despite their advantages, these works show limited performance improvement because they use a single prediction algorithm. 

Various studies have been conducted to overcome this limitation by combining multiple forecasting models. For instance, Park et al. [32] proposed a two-stage STLF model. The first stage consists of extreme gradient boosting (XGB) and RF methods for prediction, and the second stage combines their predictions using a sliding window-based multiple linear regression (MLR) model. Siridhipakul et al. [33] proposed a dual-stage attentional LSTM (DALSTM), which is a two-stage MSA electricity load-forecasting (48 time points at 30 min intervals) model based on LSTM and a dual-stage attention-based recurrent neural network (DARNN). The first stage performs MSA prediction using LSTM, and the second stage performs another MSA prediction based on the DARNN using the prediction values from the first stage and the other inputs. Moon et al. [34] proposed an MSA forecasting model using sliding window-based principal component regression (PCR), performing single-output forecasting in the first stage using four MLP models with different layers and then inputting their prediction values in the second stage. Nie et al. [35] proposed a hybrid forecasting model based on ARIMA and SVM. The model first predicted the electricity load preliminary for 24 h based on ARIMA, and then used SVM to correct the deviation from the previous prediction. Tian et al. [36] proposed a hybrid forecasting model based on LSTM and CNN. The model extracted the temporal features and local trend of the electricity load data, and used them to make final predictions. Xie et al. [37] proposed a two-stage forecasting model combining ATT-BiLSTM and MLP. First, 24 h prediction was performed based on ATT-BiLSTM, and then MLP-based prediction was performed using the result as an input.

Even though many LSTM-based methods showed strength in time series prediction, LSTM-based MSA electricity load forecasting methods have not solved the problem of performance degradation due to the increase in the prediction horizon [20]. Hence, to address this problem, we present a robust two-stage MSA forecasting model using a LightGBM-based day-ahead forecasting model and an S2S ATT-BiLSTM-based MSA forecasting model. 

## 3. Data Collection and Preprocessing

In this section, we describe the data collection and preprocessing that we performed to configure and train our forecasting model. We collected the electricity load data of buildings of a private university in Seoul, Korea at 15 min intervals from the Korea Electric Power Corporation through the i-Smart system. We classified the university buildings into four clusters based on their use and location. Cluster A consists of humanities and social sciences buildings, while clusters B and D are both science/engineering buildings and their laboratories. Finally, cluster C is dormitories and sports facilities. The data were collected for 67 months from 1 January 2015 to 31 July 2020 for clusters A, B, and C, and 58 months from 1 September 2015 to 31 July 2020 for cluster D. In addition, we considered calendar information, weather data, and historical load data as input variables to the load forecasting model. The details are described in the following subsections.

### 3.1. Weather Data

**Figure 1:**
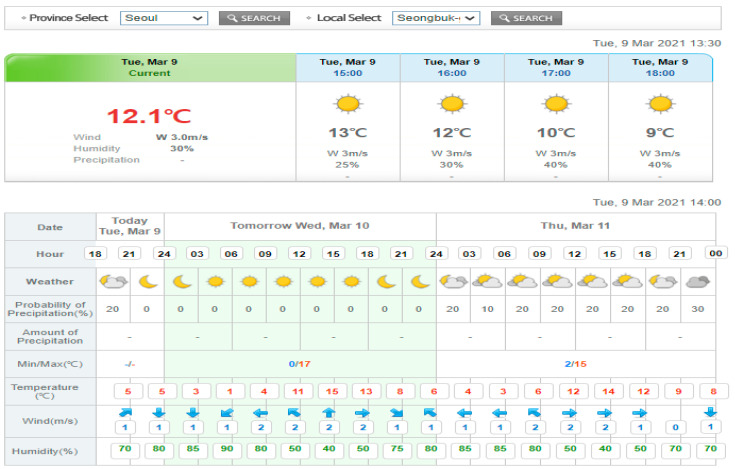
Example of Dong-Nae forecast by the Korea Meteorological Administration.

We used the weather data provided by the Korea Meteorological Administration (KMA). The KMA provides weather forecasts for most major areas, including short-term forecasts such as the Dong-Nae Forecast and mid-term forecast. Short-term forecasts provide weather forecasts every three hours for three days from the present day. As our goal is to construct a day-ahead load forecasting model, we collected weather data for the same area as the clusters using the short-term weather forecast. The short-term forecast provides sky conditions, temperature, humidity, wind speed, wind direction, precipitation, and other information. As the predictions are quite accurate, there is little difference between the forecast data and the measured data. An example of the Dong-Nae forecast is illustrated in Figure 1.

In this study, we considered four types of weather data: temperature, humidity, wind speed, and wind direction. Those weather data were provided by KMA’s short-term weather forecast as illustrated in Figure 1 [29]. Among them, weather data were provided at 3 h intervals. To construct a forecasting model at 15 min intervals, we estimated the weather data at a 15 min resolution using linear interpolation in Equation (1) [34].

(1) $$f\left(T\right)\;=\frac{d_{2}}{d_{1}+d_{2}}f\left(T_{1}\right)\;+\;\frac{d_{1}}{d_{1}+d_{2}}f\left(T_{2}\right)$$

The equation calculates the data value $f\left(T\right)$ at any point *T* between two points $T_{1}$ and $T_{2}$ where $T_{1}$ and $T_{2}$ have data values $f\left(T_{1}\right)$ and $f\left(T_{2}\right)$, respectively. Here, $d_{1}$ is the distance between T and $T_{1}$ and $d_{2}$ is the distance between T and $T_{2}$. In addition, two indices were calculated to reflect the weather the human body actually feels in the model: windchill index (WCI) for the actual temperature and discomfort index (DI) for feeling hot or cold [38]. WCI and DI are calculated using Equations (2) and (3), respectively.

(2) $$WCI=\;\left(10\sqrt{v}-v+10.5\right)\;\times \;\left(33-T_{a}\right)$$

(3) $$DI=T-0.55\times \;\left(1-0.01H\right)\;\times \;\left(T-14.5\right)$$

Here, v and $T_{a}$ indicate wind speed in m/s and temperature in degrees Celsius, respectively, and T and H represent temperature in degrees Celsius and relative humidity in %, respectively.

### 3.2. Calendar Information and Historical Electricity Load

**Figure 2:**
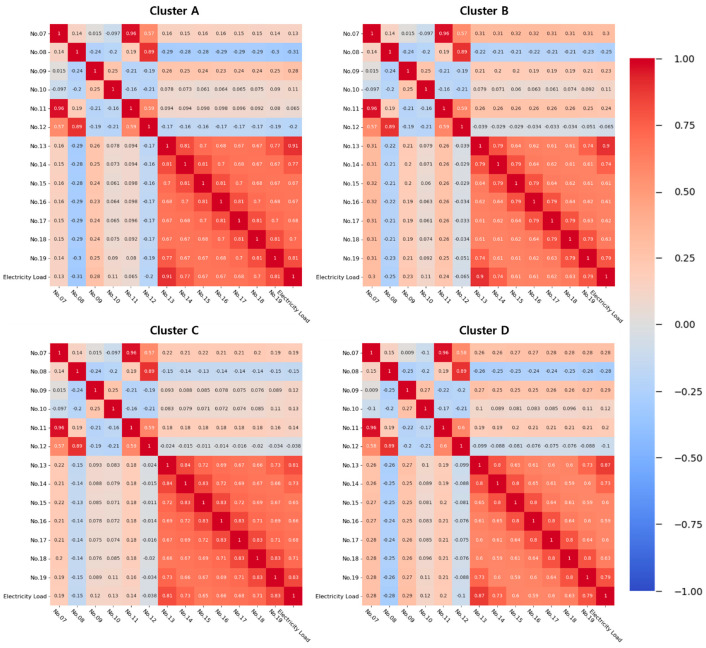
PCCs between input variables except calendar data and electricity load.

As the electricity load data are time-series data, calendar information is essential in electricity load forecasting. For calendar information, we considered months, days, hours, minutes, day of the week, and holidays, and used them as input variables of the forecasting model. Time data, including month, day, hour, and minute, are represented as numeric, and day of the week data is defined as 0 to 6 from Monday to Sunday. Holiday data are represented by one-hot encoding (i.e., “1” for holiday, otherwise “0”). We consider historical electricity load data as the input variables so that the forecasting model can reflect the recent trends in electricity load [34]. Historical electricity load data play an important role in accurate forecasting as they reveal recent electricity load patterns and trends [2]. Therefore, we selected historical electricity load data from seven days to one day before the same point as the forecast time as input variables. Table 1 presents the 19 input variables that we used to construct the forecasting model, and Figure 2 illustrates the Pearson correlation coefficients (PCCs) between the input variables and the electricity load, excluding calendar information.

In this figure, the past load of 1 day and 7 days ago shows a strong correlation with the actual electricity load. The remaining input variables are also positively related to the electrical load. 

## 4. Methodology

**Figure 3:**
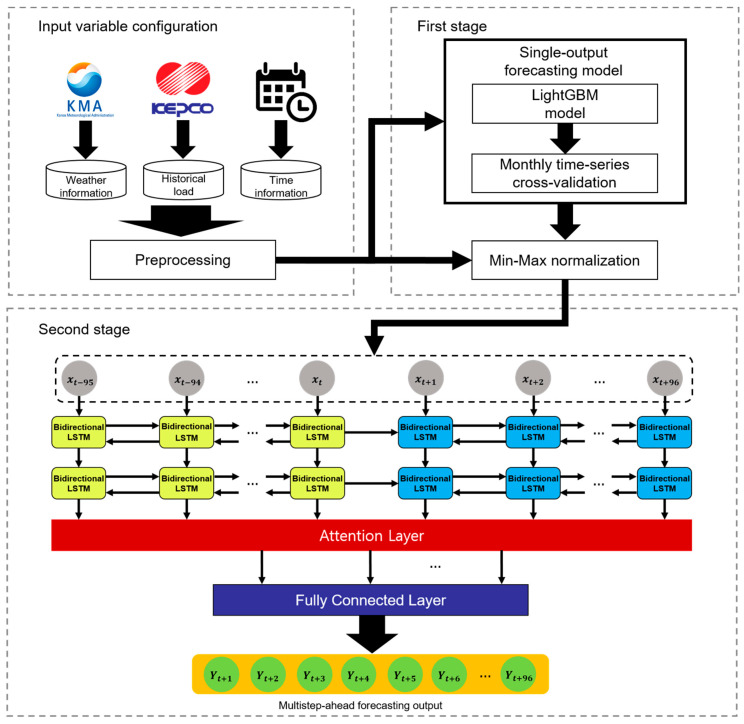
Architecture of two-stage multistep-ahead forecasting model.

In this section, we describe our two-stage MSA electricity load forecasting model. Figure 3 illustrates the overall architecture of the model. In the first stage, the LightGBM-based forecasting model performs a single-output prediction. LightGBM is a popular ensemble learning algorithm. In the second stage, a forecasting model based on bidirectional LSTM of the S2S architecture and the attention mechanism performs MSA prediction. We used data from January 2015 to December 2018 as the training set and data from January 2019 to July 2020 as the test set. The training set and test set are about 71.75% and 28.25% of the total dataset. The details are described in the following subsections.

### 4.1. Single-Output Forecasting 

In the first stage, single-output forecasting is performed using a LightGBM-based forecasting model and the forecasting values are fed into the second stage as input variables. The model was trained using four years of data. Specifically, for training, data from the first year were used as training data and TSCV was performed on the data from the next three years. Section 4.1.1 and Section 4.1.2 describe how we constructed single-output forecasting models.

#### 4.1.1. LightGBM

LightGBM is a boosting-based algorithm that allows faster and more accurate forecasting compared to other boosting and bagging algorithms [39,40]. It is based on a gradient boosting decision tree (GBDT) with gradient-based one-sided sampling and exclusive feature-bundling technologies. Unlike the traditional gradient boosting machine (GBM) tree splitting method, LightGBM uses a leafwise method to achieve higher accuracy through more complex modeling. Therefore, it is better for time series forecasting, and owing to the GBDT and leaf method, LightGBM has the advantage of low memory usage and a fast training speed. LightGBM contains many hyperparameters, of which learning rate, number of iterations, and number of leaves are closely related to forecasting accuracy. In addition, LightGBM can prevent overfitting by adjusting colsample by tree and subsample hyperparameters. LightGBM has been used for various time series forecasting tasks, such as electricity load forecasting [41,42] and wind power forecasting [43], and its single-output forecasting has been proven to be fast and accurate. As we need a fast and accurate single-output forecasting model in the first stage, we construct it using LightGBM.

#### 4.1.2. Time Series Cross-Validation

**Figure 4:**
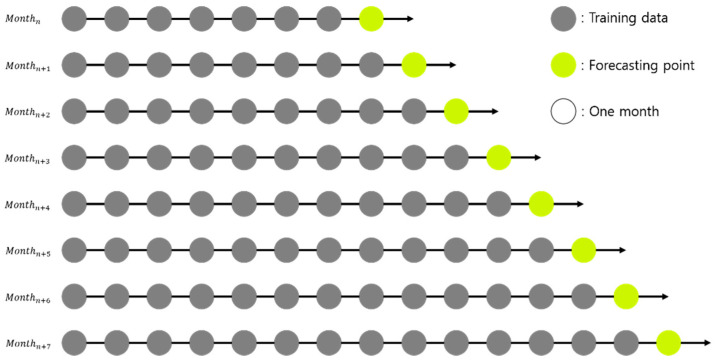
Example of monthly time-series cross-validation.

In general, to make a forecasting model, data are collected and split into a training set and a test set. The training set is used to construct the forecasting model, while the test set is used to evaluate its performance. Because single-output prediction is used as one of the inputs to the second stage forecasting model, our single-output forecasting model uses one year of training data. However, when the amount of training data is small, the accuracy decreases as the forecasting points get farther away [44]. To mitigate this concern, we used TSCV, which is a popular method when focusing on single-output forecasting in the data set when the data have time series characteristics [45]. TSCV uses all data before the forecasting point as a training set and forecasts the next forecasting point by setting it as a test set, iteratively. However, if TSCV is performed at every point in time, an enormous amount of time may be required for training and forecasting. To reduce this overhead, we used monthly TSCV, as illustrated in Figure 4.

### 4.2. Attention-BiLSTM Based MSA Forecasting

In the second stage, we constructed an MSA forecasting model of the S2S architecture based on the attention mechanism and bidirectional LSTM. In addition to the configured input variables, the model used the values of the single-output forecasting model in the first stage as its input variable. In S2S bidirectional LSTM networks, information loss could occur because the encoder compresses all the information into a fixed-length vector. To avoid this, we applied an attention mechanism to the model. 

#### 4.2.1. Bidirectional Long Short-Term Memory

**Figure 5:**
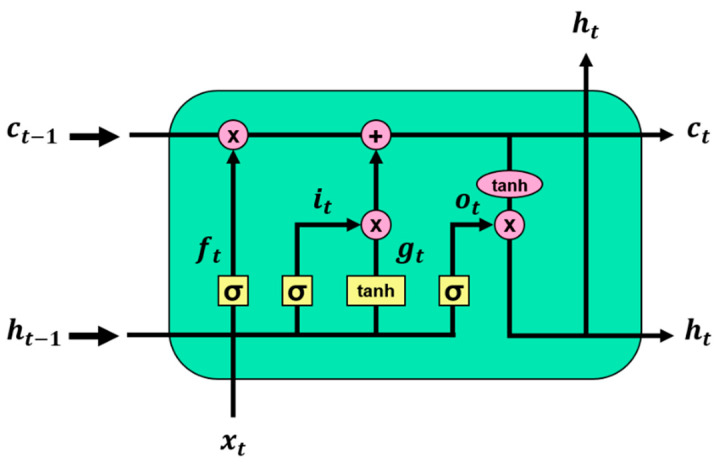
Architecture of long short-term memory.

Traditional RNNs are trained using backpropagation through time [46]. However, RNNs can exhibit the vanishing gradient problem for longer sequences of inputs [47]. LSTM [48], which was designed to mitigate this issue, is made by adding three gates to each cell of traditional RNNs. The three gates are input, forget, and output. Figure 5 illustrates the architecture of the LSTM, and the LSTM cell calculation at time t for input x is given by Equations (4)–(9). Here, *g* is an update step, and *c* and *h* indicate the cell memory state and hidden state, respectively.

(4) $$f_{t}=\sigma \;\left(W_{hf}*h_{t-1}+W_{xf}*x_{t}\right)$$

(5) $$i_{t}=\sigma \;\left(W_{hi}*h_{t-1}+W_{\varphi }*x_{t}\right)$$

(6) $$g_{t}=\tanh\;\left(W_{hg}*h_{t-1}+W_{xg}*x_{t}\right)$$

(7) $$o_{t}=\sigma \left(W_{ho}*h_{t-1}+W_{xo}*x_{t}\right)$$

(8) $$c_{t}=f_{t}⊙c_{t-1}+i_{t}⊙g_{t}$$

(9) $$h_{t}=o_{t}⊙\tanh\;\left(c_{t}\right)$$

**Figure 6:**
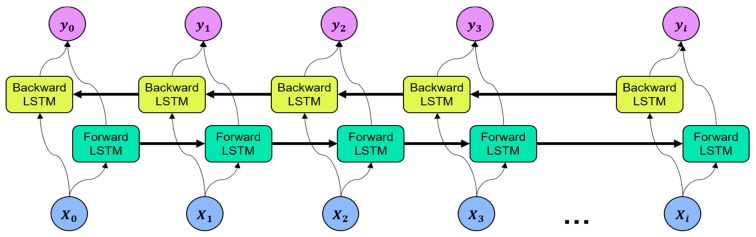
Architecture of bidirectional long short-term memory.

LSTM stores the information of the input data in a hidden layer by adding the concept of time series. However, as the input information is stacked in the time sequence at the hidden layer, the most recent input information is reflected in the result [49]. Bidirectional LSTM (BiLSTM) is an extended version of the traditional LSTM that considers past and future states to improve forecasting performance [50]. BiLSTM processes data into two networks, i.e., the forward LSTM and the backward LSTM, and the outputs of the two networks are merged at each time step. The architecture of the BiLSTM is illustrated in Figure 6.

#### 4.2.2. Sequence-to-Sequence Recurrent Neural Networks

**Figure 7:**
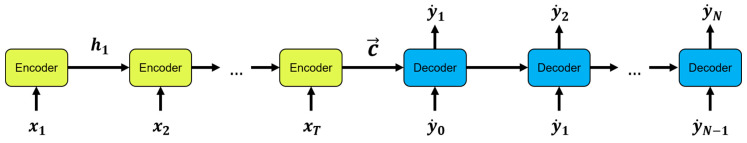
Sequence-to-Sequence recurrent neural network.

An S2S RNN contains two RNNs, an encoder and a decoder, as shown in Figure 7. The general idea is to pass an input sequence vector $x_{1},\;x_{2},\;\ldots ,\;x_{T}$ one time step at a time to the encoder RNN to obtain a context vector. A common approach is to use an encoder RNN, as given by Equations (10) and (11).

(10) $$h_{j}=f^{*}\left(x_{j},h_{j-1}\right)$$

(11) $$\vec{c}=q\left(\{h_{1},\ldots ,h_{T}\right)$$

Here, $h_{j}$ is the hidden state at time j, and $f^{*}$ and q are nonlinear functions [51]. $\vec{c}$ is generated from a sequence of hidden states. The context vector is an encoded representation of the input sequence, passed to the decoder RNN, which extracts information at each unraveled time step to obtain the output sequence ${\dot{y}}_{1},\;\ldots ,{\dot{y}}_{N}$. The S2S output is obtained using Equation (12).

(12) $${\dot{y}}_{N}=g^{*}\left({\dot{y}}_{i-1},\;\ldots ,h_{i-1}^{*}\right)$$

Here, $h_{i}^{*}$ is the hidden state of the decoder at time i and $g^{*}$ is a nonlinear function. ${\dot{y}}_{0}$ is the context value (derived value from $\vec{c}$) used as the initial input to the decoder. The S2S RNN can enhance the continuous sequence forecasting and the temporal dimensions of the inputs and outputs. This can improve the performance of electricity load forecasting.

#### 4.2.3. Attention Mechanism

In a model of S2S architecture, the encoder compresses all the information of the input sequence into a single context vector. For longer input sequences, the decoder has difficulties extracting valuable information from this single vector. To address this issue, attention mechanisms have been devised. Bahdanau et al. [18] and Luong et al. [19] each added attention mechanisms to the S2S model for machine translation. Machine translation is a very different task from predicting electricity loads. Nevertheless, various studies [20,30] have shown that the attention mechanism benefits electricity load forecasting. Our model adopts the Bahdanau attention mechanism (BA) for electricity load forecasting. The S2S model with BA first obtains the hidden state of the current decoder at time i using Equation (13). The forecasted value is then obtained using Equation (14).

(13) $$h_{i}^{b}=f^{b}\left(\left[{\dot{y}}_{i-1};c_{i}^{b}\right]\;+\;h_{i-1}^{b}\right)$$

(14) $${\dot{y}}_{i}=g^{b}\left({\dot{y}}_{i-1},c_{i}^{b},h_{i}^{b}\right)$$

Here, *b* denotes the variable used in BA, and *i* and *j* denote the decoder and encoder variables, respectively. In the equation, $f^{b}$ takes the previous output ${\dot{y}}_{i-1}$, attention context vector $c_{i}^{b},$ and previous hidden state $h_{i-1}^{b}$. $f^{b}$ and $g^{b}$ are the LSTM cell and nonlinear function, respectively. Each encoder output $h_{j}$ contains information for the j-th part of the input sequence. The vector $c_{i}^{b}$ is calculated as the weighted sum of the encoder outputs, as shown in Equation (15).

(15) $$c_{i}^{b}=\overset{T}{\underset{j=1}{\sum }}\alpha_{ij}^{b}h_{j}$$

The attention weight $\alpha_{ij}^{b}$ of each encoder output *j* is calculated using Equation (16).

(16) $$\alpha_{ij}^{b}=\frac{\exp\left(e_{ij}^{b}\right)}{\sum_{k=1}^{T}\exp\left(e_{ik}^{b}\right)}$$

The attention scores $e_{ij}^{b}$ are calculated using Equation (17).

(17) $$e_{ij}^{b}=S\left(h_{i-1}^{b},h_{j}\right)$$

Here, *S* is an alignment model that scores how well the inputs around time j and the output at time i match. Attention weight $\alpha_{ij}^{b}$ and attention score $e_{ij}^{b}$ reflect the importance of each encoder output $h_{j}$ when generating the next hidden state $h_{i}^{b}$ and forecasting value ${\dot{y}}_{i}$. This allows the decoder to pay attention to the important parts of the input sequence.

In the second stage, we combine the attention mechanism with the Sequence-to-Sequence BiLSTM and construct an S2S ATT-BiLSTM-based MSA forecasting model that performs MSA electricity load forecasting at 96 time points at 15 min intervals. To construct the BiLSTM network, we considered several hyperparameters. The input layer of the BiLSTM model consists of 20 nodes, and the hidden layer consists of 15 nodes per layer by applying 2/3 of the input layer and the size of the output layer [34]. We used two layers for the number of hidden layers. The fully connected layer activation function uses a rectified linear unit (ReLU) to solve the gradient vanishing problem [52]. Huber loss [53] was used as the loss function, and adaptive moment estimation (Adam) [54] was used as the optimization algorithm. The learning rate and epochs were set to 0.001 and 350.

## 5. Results and Discussion

**Figure 8:**
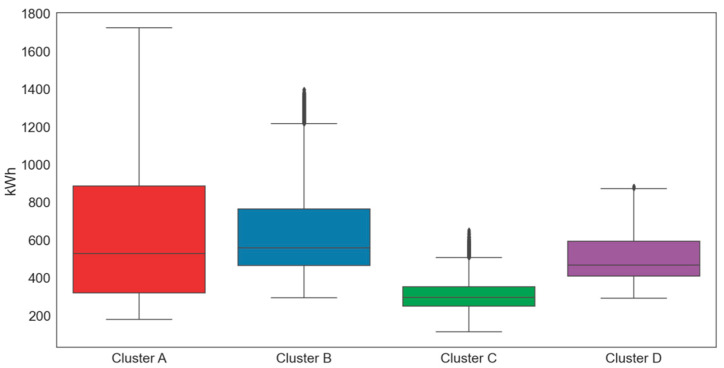
Boxplots by cluster (kWh).

As our forecasting model is composed of two stages, each with a different predictive model, we performed comparative experiments for each model. In the experiments, we used electricity load data collected from four building clusters of a private university in Seoul, Korea, at 15 min intervals through the i-Smart system. The clusters were determined based on their use and location. We first investigated the electric load characteristics of four clusters via box plot and various statistical analyses, as presented in Figure 8 and Table 2. For statistical analysis, we used the descriptive statistical data analysis tool of Excel.

To reflect all data with the same degree of importance, the input data were preprocessed by min–max normalization, defined by Equation (18). In the equation, x represents the original data, and $x_{min}$ and $x_{max}$ represent the minimum and maximum values of the original data, respectively. Finally, all the values are normalized to between 0 and 1.

(18) $$x_{norm}=\frac{x-x_{min}}{x_{max}-x_{min}}$$

To evaluate the forecasting performance of the proposed model, we used four metrics: mean absolute error (MAE), mean absolute percentage error (MAPE), root mean square error (RMSE), and normalized root mean square error (NRMSE)**,** as given in Equations (19)–(22). Here, $A_{t}$ and $F_{t}$ represent the actual and forecasted values, respectively, at time t. n indicates the number of observations, and $\bar{A}$ represents the mean of the actual values.

(19) $$MAE=\frac{1}{n}\sum_{t\;=\;1}^{n}\left|A_{t}-F_{t}\right|$$

(20) $$MAPE=\frac{100}{n}\sum_{t\;=\;1}^{n}\left|\frac{\left|A_{t}-F_{t}\right|}{A_{t}}\right|$$

(21) $$RMSE=\sqrt{\frac{\sum_{t\;=\;1}^{n}{\left(F_{t}-A_{t}\right)}^{2}}{n}}$$

(22) $$NRMSE=\frac{\sqrt{\frac{\sum_{t\;=\;1}^{n}{\left(F_{t}\;-\;A_{t}\right)}^{2}}{n}}}{\bar{A}}\times 100$$

In the experiment, Intel (R) Core (TM) i7-10700 CPU, Samsung 32 G DDR4 memory, and NVIDIA Geforce GTX 3090ti were used, and the operating system was Windows 10. Electricity load forecasting of our proposed model was performed in Python 3.7. The ensemble learning models in the first stage were constructed using scikit-learn (v.0.22.1) and tuned using a grid search [55]. The RNN-based model in the second stage was constructed using Pytorch 1.7.1 [56]. The experiments and their results for each stage are illustrated in the following subsections.

### 5.1. Single-Output Forecasting Results

In the first experiment, we compared our single-output forecasting model with other popular machine learning models. For fair comparison, TSCV was also applied to the machine learning-based forecasting models. Table 3 summarizes the hyperparameter values determined for the models by the grid search. In total, 365 days of data were used for initial training, while 1674 days in clusters A, B, and C, and 1430 days in cluster D, were used as test data. The comparative experimental results of single-output forecasting models are presented in Table 4.

The experimental results show that the LightGBM model outperformed other forecasting models in all metrics in most clusters. Therefore, we used the LightGBM forecasting values as the new input variable for the second stage. Table 5 presents the PCC between the single-output forecasting results of LightGBM and the actual electricity loads, which indicates a strong correlation between them. Therefore, we use a single-output prediction value for the second stage input to improve the MSA forecasting performance.

### 5.2. Multistep-Ahead Forecasting Results

In order to evaluate the performance of the proposed model, we carried out extensive experiments with various MSA forecasting models. As mentioned earlier, in the experiments, we used data from January 2016 to December 2018 of clusters A, B, and C and data from September 2016 to December 2018 of cluster D as the training set, and data from January 2019 to July 2020 as the test set. To evaluate the validity of our forecasting model, we compared it with basic deep learning, ensemble learning-based MSA forecasting models, and other models including ATT-GRU [30], DALSTM [33], and COSMOS [34]. The ensemble learning-based models require all input variables for 96 forecasting time points for MSA forecasting. Therefore, we used 1824 input variables (i.e., 19 input variables × 96 forecasting points). The ensemble learning-based MSA forecasting models perform predictions on multiple outputs using the MultiOutputRegressor module in scikit-learn. The hyperparameter configurations of comparative forecasting models are presented in Table 6.

**Figure 9:**
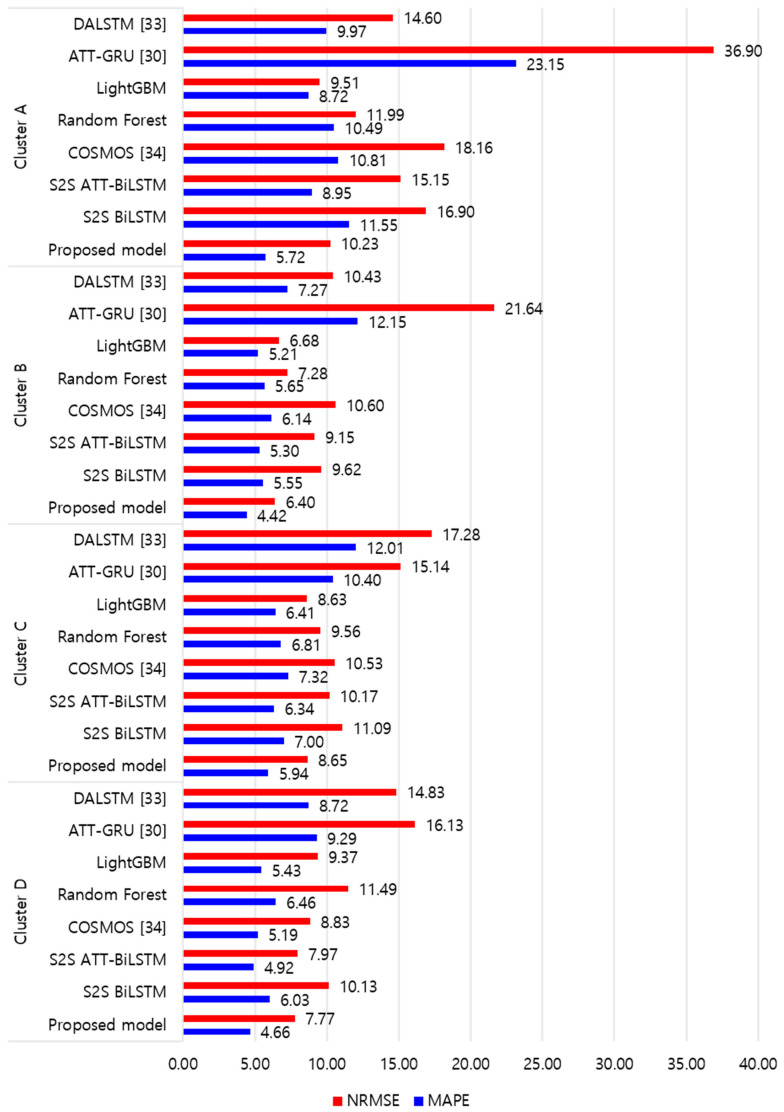
Average of NRMSE and MAPE for each model (%).

**Figure 10:**
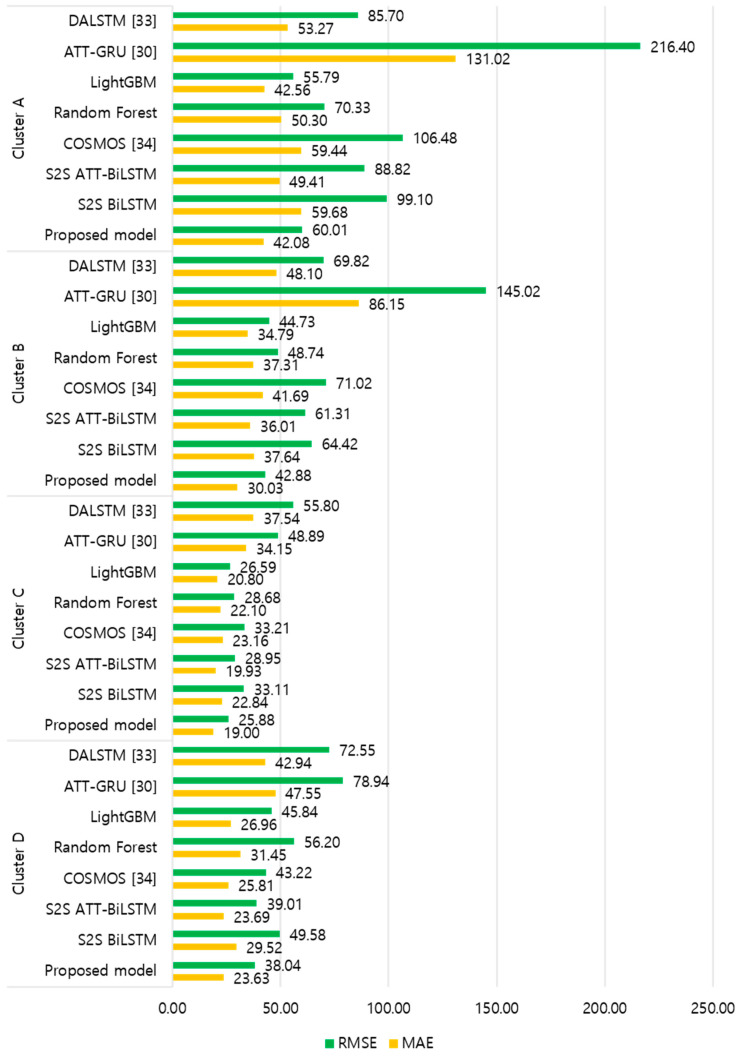
Average of RMSE and MAE for each model (kWh).

**Figure 11:**
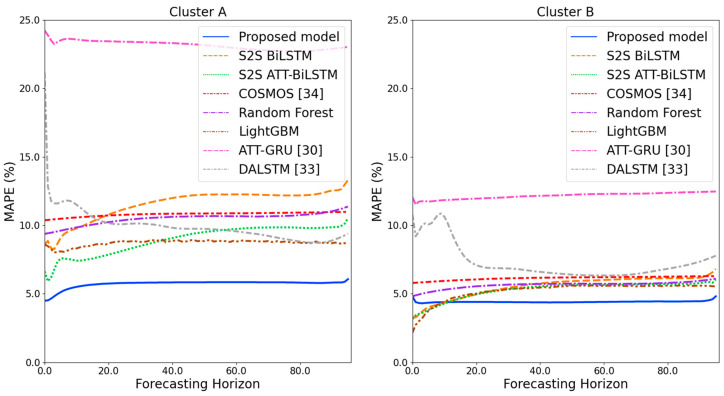
MAPE results of clusters A and B over the entire forecasting horizon in (%).

**Figure 12:**
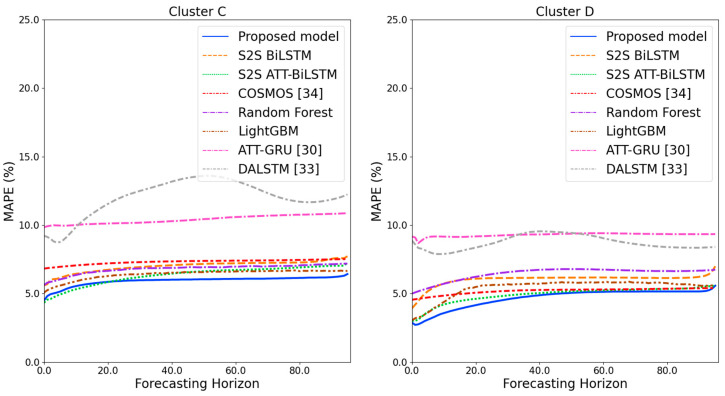
MAPE results of clusters C and D over the entire forecasting horizon (%).

**Figure 13:**
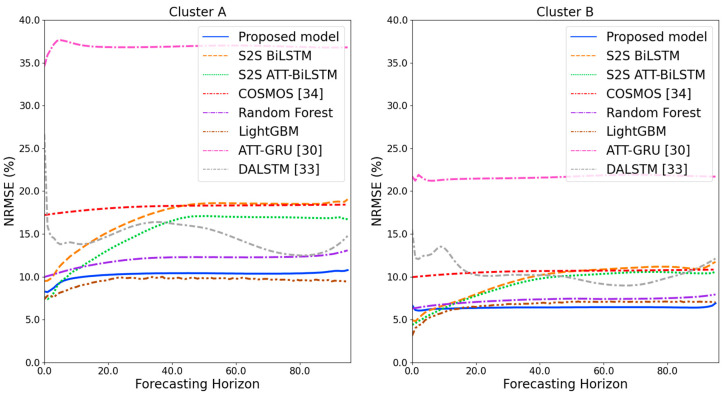
NRMSE results of clusters A and B over the entire forecasting horizon (%).

**Figure 14:**
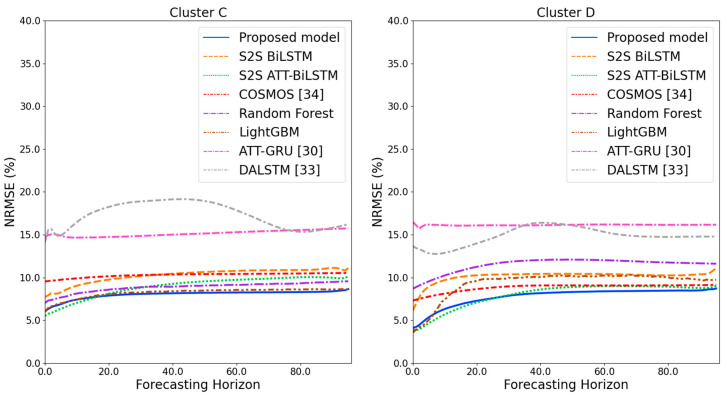
NRMSE results of clusters C and D over the entire forecasting horizon (%).

**Figure 15:**
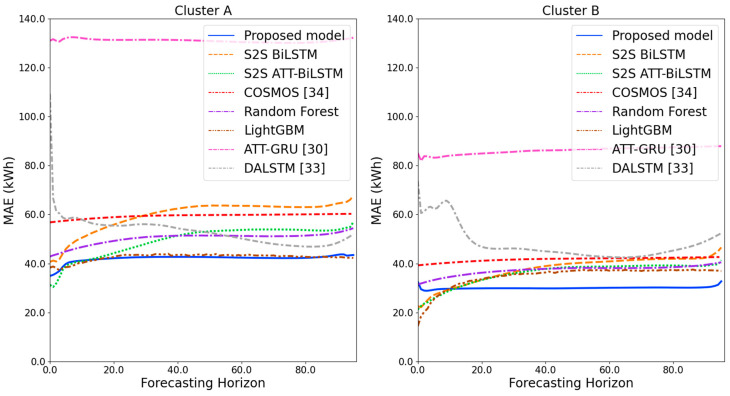
MAE results of clusters A and B over the entire forecasting horizon (kWh).

**Figure 16:**
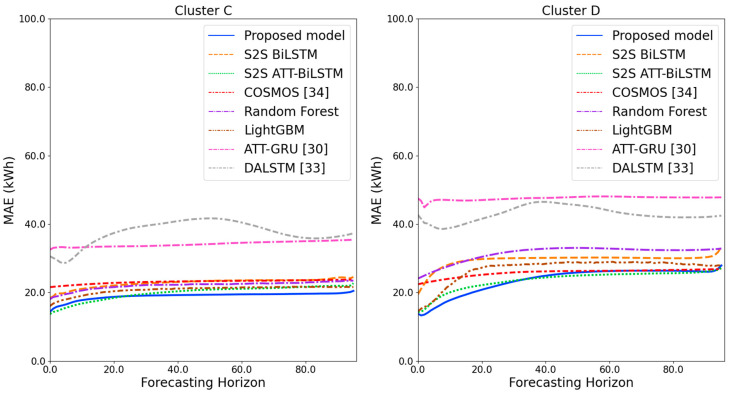
MAE results of clusters C and D over the entire forecasting horizon (kWh).

**Figure 17:**
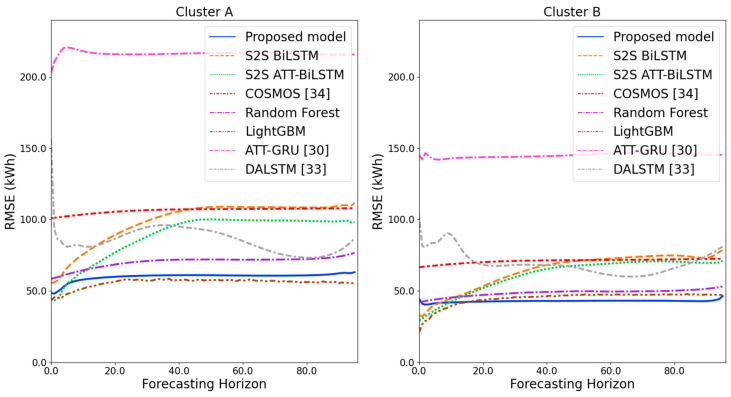
RMSE results of clusters A and B over the entire forecasting horizon (kWh).

**Figure 18:**
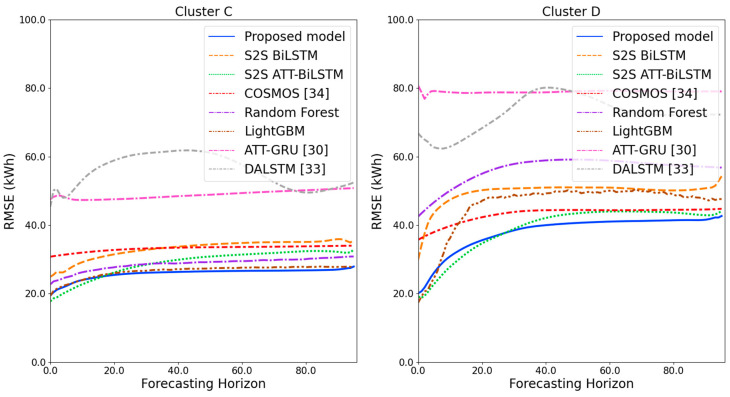
RMSE results of clusters C and D over the entire forecasting horizon (kWh).

Figure 9 and Figure 10 illustrate the average of the four evaluation metrics of the forecasting models for each cluster over the entire forecasting horizon. For instance, we can see that the proposed model achieved the best NRMSE and MAPE across all clusters, with one exception, as shown in Figure 9. That is, the LightGBM-based forecasting model achieved the best NRMSE in cluster A with a narrow margin compared to the proposed model. Similarly, the proposed model achieved the best performance for MAE and RMSE across all clusters, with one exception, as shown in Figure 10. The LightGBM-based forecasting model achieved the best RMSE in cluster A with a narrow margin compared to the proposed model. Furthermore, from the evaluation results of S2S BiLSTM, S2S ATT-BiLSTM, and the proposed model, it can be seen that attention and two-stage forecasting can improve the forecasting performance. Figure 11, Figure 12, Figure 13, Figure 14, Figure 15, Figure 16, Figure 17 and Figure 18 show the trends of the metric values of the forecasting models over the entire forecasting horizon for clusters A, B, C and D. In the figures, the *X*-axis represents forecasting time points, and the *Y*-axis represents their forecasting errors.

Figure 11 and Figure 12 illustrate the MAPE results for the four clusters over the entire forecasting horizon. They show that the proposed model outperforms the other models for all clusters. Similarly, Figure 13 and Figure 14 show that the proposed model achieves the best NRMSE for all clusters except cluster A. On the other hand, Figure 15 and Figure 16 present the MAE results for the four clusters, and the proposed model shows the best performance out of all clusters. Lastly, Figure 17 and Figure 18 illustrate the RMSE results, and the proposed model shows the best performance for all clusters except cluster A.

From the figures, we can see that the proposed model gives very stable and high-quality prediction performance in terms of four evaluation metrics over the entire forecasting horizon for all clusters, compared to other forecasting models. On the other hand, ATT-GRU [30] shows poor prediction performance because it cannot reflect a lot of information due to using a single network. Similarly, DALSTM [33] has poor forecasting accuracy in the second stage because the DARNN model is unsuitable for multistep-ahead forecasting. On the other hand, COSMOS [34] showed stable performance, as it used only the predicted values as inputs for MSA forecasting. However, its performance was greatly affected by the first-stage prediction. The only exception was the LightGBM-based forecasting model, which gave slightly better average RMSE and NRMSE for cluster A only. Furthermore, we compared the proposed model with the S2SATT-BiLSTM-based model to show that the proposed model combined with a single-output prediction can reliably predict over the entire forecasting horizon. In addition, we compared the S2S ATT-BiLSTM- and S2S BiLSTM-based models to verify the influence of attention mechanisms on prediction accuracy.

## 6. Conclusions

In this paper, we proposed a robust two-stage MSA forecasting model that combines a single-output forecasting model and a MSA forecasting model. To show the effectiveness of the proposed model, we conducted extensive comparative experiments with other popular forecasting models using electricity load data of four types of building clusters at a private university. Overall, the experimental results showed that the forecasting performance can be improved by combining the two models and by using an attention mechanism. Nevertheless, the proposed model cannot explain the evidence of the output properly. Hence, in future work, we plan to implement explainable artificial intelligence through attention score analysis of attention mechanism. We will also investigate how to optimize hyperparameters to improve forecasting performance.

## Figures and Tables

**Table 1:** List of input variables for the proposed model.

Input Variable Identifier	Description (Type)	Input Variable Identifier	Description (Type)
No.01	Month (numeric)	No.11	Windchill index (numeric)
No.02	Day (numeric)	No.12	Discomfort index (numeric)
No.03	Hour (numeric)	No.13	D−7 same point load (numeric)
No.04	Min (numeric)	No.14	D−6 same point load (numeric)
No.05	Day of the week (numeric)	No.15	D−5 same point load (numeric)
No.06	Holiday (binary)	No.16	D−4 same point load (numeric)
No.07	Temperature (numeric)	No.17	D−3 same point load (numeric)
No.08	Humidity (numeric)	No.18	D−2 same point load (numeric)
No.09	Wind speed (numeric)	No.19	D−1 same point load (numeric)
No.10	Wind direction (numeric)		

**Table 2:** Statistical analysis of electricity load data by cluster (kWh).

	Cluster A		Cluster B		Cluster C		Cluster D	
	Training Set	Test Set	Training Set	Test Set	Training Set	Test Set	Training Set	Test Set
Mean	656.499	586.247	623.109	670.012	302.790	322.904	515.166	489.349
Standard error	0.954	1.418	0.587	0.929	0.205	0.323	0.35	0.515
Median	553.4	462.7	543.8	593.3	292.3	311.8	478.2	447.3
Mode	271.2	265.9	454.1	518.4	259.9	275.8	413.4	414.9
Standard deviation	357.686	333.674	220.187	218.575	76.902	76.172	119.92	121.328
Sample variance	127,939.5	111,338.3	48,482.45	47,775.23	5914.042	5802.298	14,380.87	14,720.47
Kurtosis	−0.721	−0.758	0.352	0.434	0.375	0.233	−0.597	−0.225
Skewness	0.676	0.736	1.076	1.096	0.689	0.716	0.673	0.871
Range	1529.7	1350.2	1104	1017.1	541.8	476.9	586.8	556.5
Minimum	195.4	181.4	296.2	383.2	114.1	130.7	300.6	292.5
Maximum	1725.1	1531.6	1400.2	1400.3	655.9	607.6	887.4	849
Sum	92,204,063	32,417,138	87,514,440	37,049,022	42,526,339	17,855,317	60,336,352	27,059,063
Count	140,448	55,296	140,448	55,296	140,448	55,296	117,120	55,296

**Table 3:** Selected hyperparameters for each single-output forecasting model. Selected values are bold.

Model	Cluster A	Cluster B	Cluster C	Cluster D
LightGBM	Learning rate:	Learning rate:	Learning rate:	Learning rate:
	0.01, 0.05, **0.1**	0.01, 0.05, **0.1**	0.01, 0.05, **0.1**	0.01, 0.05, **0.1**
	No. of iterations: 500, **1000**	No. of iterations: 500, **1000**	No. of iterations: 500, **1000**	No. of iterations: 500, **1000**
	No. of leaves: **64**	No. of leaves: **64**	No. of leaves: **64**	No. of leaves: **64**
	Subsample: **0.5**, 1.0	Subsample: **0.5**, 1.0	Subsample: **0.5**, 1.0	Subsample: **0.5**, 1.0
XGBoost	Learning rate: **0.01**, 0.05, 0.1	Learning rate: **0.01**, 0.05, 0.1	Learning rate: 0.01, **0.05**, 0.1	Learning rate: 0.01, **0.05**, 0.1
	No. of iterations: **500**, 1000	No. of iterations: **500**, 1000	No. of iterations: **500**, 1000	No. of iterations: 500, **1000**
	Subsample: 0.5, **1.0**	Subsample: 0.5, **1.0**	Subsample: **0.5**, 1.0	Subsample: **0.5**, 1.0
	Colsample by tree:	Colsample by tree:	Colsample by tree:	Colsample by tree:
	0.5, **1.0**	0.5, **1.0**	0.5, **1.0**	0.5, **1.0**
NGBoost	No. of iterations: 500, **1000**, 1500	No. of iterations: 500, **1000**, 1500	No. of iterations: 500, 1000, **1500**	No. of iterations: 500, 1000, **1500**
Random Forest	No. of trees: 64, **128**	No. of trees: 64, **128**	No. of trees: 64, **128**	No. of trees: 64, **128**
	Random state: 32, **64**	Random state: 32, **64**	Random state: 32, **64**	Random state: 32, **64**
MLP	No. of layers: 4, 5, **6**, 7	No. of layers: 4, 5, **6**, 7	No. of layers: 4, 5, **6**, 7	No. of layers: 4, 5, **6**, 7
	Activation function: **ReLU**	Activation function: **ReLU**	Activation function: **ReLU**	Activation function: **ReLU**
	Optimizer: **Adam**	Optimizer: **Adam**	Optimizer: **Adam**	Optimizer: **Adam**
	Learning rate: **0.001**	Learning rate: **0.001**	Learning rate: **0.001**	Learning rate: **0.001**

**Table 4:** The comparative experimental results of single-output forecasting models.

Evaluation Metric	Model	Cluster A	Cluster B	Cluster C	Cluster D
MAPE (%)	LightGBM	7.01	4.74	6.98	4.98
	MLP	12.06	7.03	7.61	5.67
	RF	7.53	4.98	7.24	5.38
	XGBoost	7.22	5.12	7.52	5.29
	NGBoost	9.64	5.44	7.73	5.90
MAE (kWh)	LightGBM	40.71	32.00	22.18	23.47
	MLP	68.59	45.95	23.10	27.30
	RF	43.81	34.09	23.46	25.49
	XGBoost	41.81	32.81	24.76	24.81
	NGBoost	54.25	37.04	24.85	29.21
RMSE (kWh)	LightGBM	61.31	49.01	30.81	36.26
	MLP	119.07	75.39	32.5	41.11
	RF	67.2	54.73	32.89	40.51
	XGBoost	63.58	48.34	34.39	37.16
	NGBoost	82.05	59.1	34.43	44.17
NRMSE (%)	LightGBM	9.37	7.63	9.78	7.25
	MLP	18.91	11.74	10.32	8.22
	RF	10.67	8.52	10.45	8.10
	XGBoost	10.09	7.53	10.92	7.43
	NGBoost	13.03	9.21	10.93	8.83

**Table 5:** PCC between single-output forecasting and actual electricity loads.

Cluster A	Cluster B	Cluster C	Cluster D
0.984	0.978	0.923	0.957

**Table 6:** Selected hyperparameters for each multistep-ahead forecasting model.

Model	Package	Selected Hyperparameters
LightGBM	LightGBM Scikit-learn	Learning rate: 0.05
		No. of iterations: 1000
		No. of leaves: 32
		Subsample: 0.5
Random Forest	Scikit-learn	No. of trees: 128
		Random state: 64
S2S BiLSTM	Pytorch	No. of hidden nodes: 15
		No. of hidden layers: 2
		Activation function: ReLU
		Optimizer: Adam
		Learning rate: 0.001
		No. of epochs: 350
S2S ATT-BiLSTM	Pytorch	No. of hidden nodes: 15
		No. of hidden layers: 2
		Activation function: ReLU
		Optimizer: Adam
		Learning rate: 0.001
		No. of epochs: 350
ATT-GRU [30]	Pytorch	No. of hidden nodes: 15
		No. of hidden layers: 2
		Activation function: SELU
		Optimizer: Adam
		Learning rate: 0.001
		No. of epochs: 150
DALSTM [33] Stage 1: LSTM Stage 2: DARNN	Pytorch	LSTM
		No. of hidden nodes: 15
		No. of hidden layers: 2
		Activation function: ReLU
		Optimizer: Adam
		Learning rate: 0.001
		No. of epochs: 350
		DARNN
		No. of hidden nodes: 64
		Time steps: 96
		Optimizer: Adam
		Learning rate: 0.001
		No. of epochs: 150
COSMOS [34] Stage 1: MLP Stage 2: PCR	Scikit-learn	MLP
		No. of hidden nodes: 15
		No. of hidden layers: 4, 5, 6, 7
		Activation function: ReLU
		Optimizer: Adam
		Learning rate: 0.001
		No. of epochs: 150
		PCR
		Principal components: 1
		Sliding window size: 672

## Data Availability

Not applicable.

## Abbreviations

The following abbreviations are used in this manuscript

ICT	information and communications technology
STLF	Short-term load forecasting
AI	artificial intelligence
MSA	multistep-ahead
RNN	recurrent neural network
LSTM	long short-term memory
GRU	gated recurrent unit
S2S	sequence-to-sequence
ANN	artificial neural network
ARIMA	autoregressive integrated moving average
MLR	multiple linear regression
PCR	principal component regression
PCC	Pearson correlation coefficient
WCI	windchill index
GBM	gradient boosting machine
ReLU	rectified linear unit
MAE	mean absolute error
RMSE	root mean square error
NN	neural network
DARNN	dual-stage attention-based recurrent neural network
ATT-GRU	attention-based gated recurrent unit
LightGBM	light gradient boosting machine
TSCV	Time series cross-validation
BiLSTM	bidirectional long short-term memory
ATT-BiLSTM	bidirectional long short-term memory with attention mechanism
SVR	support vector regression
RF	random forest
FIR	fuzzy inductive reasoning
MLP	multilayer perceptron
CNN	convolutional neural network
XGB	extreme gradient boosting
ML	machine learning
KMA	Korea meteorological administration
DI	discomfort index
GBDT	gradient boosting decision tree
BA	Bahdanau attention mechanism
Adam	adaptive moment estimation
MAPE	mean absolute percentage error
NRMSE	normalized root mean square error
RICNN	recurrent inception convolution neural network
DALSTM	dual-stage attentional long short-term memory
COSMOS	combination of short-term load forecasting models using a stacking ensemble approach
